# Mental Health Interventions in Special Education Schools

**DOI:** 10.1192/j.eurpsy.2025.126

**Published:** 2025-08-26

**Authors:** L. M. Sevilla Cermeño

**Affiliations:** Department of Child and Adolescent Psychiatry, Hospital General Universitario Gregorio Marañón, Madrid, Spain

## Abstract

**Abstract:**

Children in special education schools are particularly susceptible to developing mental health issues. Specifically, it is estimated that 40% of individuals with intellectual disabilities have a comorbid mental disorder diagnosis (1). However, access to mental health services for patients with intellectual disabilities remains far below expectations. Numerous barriers impede this access, including a lack of coordination between professionals and service providers responsible for their care (2).

Therefore, interventions within special education schools, promoting early detection and intervention for psychopathology and facilitating coordination between educational and healthcare services, are critically important.

We present an innovative mental health care resource designed for special education schools in the Community of Madrid, Spain. This initiative combines multi-disciplinary expertise with flexible, hybrid care delivery to ensure accessibility for students across 14 public schools. The team consists of a psychiatrist, a clinical psychologist, and a mental health nurse who provide both in-person and remote assistance, addressing the psychopathology exhibited by their students.

Preliminary results suggest that this intervention has the potential to improve early detection rates of mental health issues and foster better coordination between education and healthcare systems. This model could serve as a blueprint for similar programs worldwide, addressing significant gaps in mental health care for children with intellectual disabilities.

**References:**

(1) Cooper, S. A., Smiley, E., Morrison, J., Williamson, A., & Allan, L. (2007). Mental ill-health in adults with intellectual disabilities: prevalence and associated factors. The British Journal of Psychiatry, 190, 27–35. https://doi.org/10.1192/bjp.bp.106.022483

(2) Whittle, E. L., Fisher, K. R., Reppermund, S., Lenroot, R., & Trollor, J. (2017). Barriers and enablers to accessing mental health services for people with intellectual disability: A scoping review. Journal of Mental Health Research in Intellectual Disabilities, 11(1), 69–102. https://doi.org/10.1080/19315864.2017.1408724

**Disclosure of Interest:**

None Declared

